# Steering decision making by terminology: oligometastatic versus argometastatic

**DOI:** 10.1038/s41416-022-01879-3

**Published:** 2022-06-17

**Authors:** Petr Szturz, Jan B. Vermorken

**Affiliations:** 1 Medical Oncology, Department of Oncology, University of Lausanne (UNIL) and Lausanne University Hospital (CHUV), Lausanne, Switzerland; 2 Faculty of Medicine and Health Sciences, University of Antwerp, Antwerp, Belgium; 3 Department of Medical Oncology, Antwerp University Hospital, Edegem, Belgium

**Keywords:** Cancer models, Metastasis

## Abstract

Allowing selected patients with few distant metastases to undergo potentially curative local ablation, the designation “oligometastatic” has become a widely popular concept in oncology. However, accumulating evidence suggests that many of these patients harbour an unrecognised microscopic disease, leading either to the continuous development of new metastases or to an overt polymetastatic state and questioning thus an indiscriminate use of potentially harmful local ablation. In this paper, reviewing data on oligometastatic disease, we advocate the importance of identifying a true oligometastatic disease, characterised by a slow speed of development, instead of relying solely on a low number of lesions as the term “oligometastatic” implies. This is particularly relevant in clinical practice, where terminology has been shown to influence decision making. To define a true oligometastatic disease in the context of its still elusive biology and interaction with the immune system, we propose using clinical criteria. As discussed further in the paper, these criteria can be classified into three categories involving a low probability of occult metastases, low tumour growth rate and low tumour burden. Such cases with slow tumour-cell shedding and slow proliferation leave a sufficiently broad window-of-opportunity to detect and treat accessible lesions, increasing thus the odds of a cure.

In 1995, Hellman and Weichselbaum summarised available evidence on local ablation of distant metastases, deducing the concept of oligometastases as an intermediate state between a localised tumour and a widespread metastatic disease [1]. The authors emphasised the principal condition of a limited number and sites of metastases (from Greek “oligos” meaning few) that could offer some patients a potentially curative therapeutic opportunity, but no specific diagnostic criteria were provided. Also ignited by an accelerated availability and use of new methods of local ablation including stereotactic ablative body radiotherapy (SABR) and radiofrequency ablation, the next 25 years were marked by an exponential rise of interest in oligometastatic disease [2]. Emerging prospective clinical trials, both single-arm and randomised, have often relied on the number of distant metastases not surpassing five [3]. This criterion has been adopted in the recent consensus document of the European Society for Radiotherapy and Oncology (ESTRO) and American Society for Radiation Oncology (ASTRO), complying with the need for standardisation to meaningfully advance scientific research [4]. However, mounting evidence, also originating from the above-mentioned trials, has pointed to the drawbacks of a definition based on snapshot imaging as will be further discussed in this paper.

The STOMP (Surveillance or Metastasis-Directed Therapy for Oligometastatic Prostate Cancer Recurrence) trial was a phase II study randomly assigning asymptomatic prostate cancer patients with a biochemical recurrence after primary treatment to either surveillance or metastasis-directed therapy (surgery or SABR). The patients had to have a maximum of three extracranial lesions detected on choline positron emission tomography/computed tomography (PET/CT) imaging. Although the study met its primary endpoint of median androgen deprivation therapy-free survival improvement (13 versus 21 months, hazard ratio, 0.60; 80% CI, 0.40–0.90; log-rank *P* = 0.11), no difference was observed in the rates of polymetastatic progression (55% versus 61%) [5]. Similarly, the landmark SABR-COMET (Stereotactic Ablative Radiotherapy for the Comprehensive Treatment of Oligometastases) trial, which was the first study to investigate the impact of local ablation of oligometastases on overall survival, randomised 99 patients with different types of primary tumours and five or fewer metastases to either palliative standard of care alone or complemented with SABR. The addition of local intervention enhanced median overall survival from 28 to 41 months (hazard ratio, 0.57; 95% CI, 0.30–1.10; log-rank *P* = 0.090), but the proportion of patients presenting with new metastases was almost identical in both arms (58% versus 60%), possibly owing to subclinical dissemination [6].

The unrecognised microscopic disease could have also contributed to the results of the following study focusing on a surgical approach. The phase III PulMiCC (Pulmonary Metastasectomy versus Continued Active Monitoring in Colorectal Cancer) trial explored if there was a benefit of pulmonary metastasectomy in colorectal cancer patients over active surveillance. Despite being prematurely terminated after enrolling only 65 patients due to difficulties in accrual, the two arms were well-balanced and the trial provocatively demonstrated a lower-than-expected difference in the estimated 5-year overall survival which was 38% for metastasectomy (1–5 lesions) versus 29% for surveillance [7]. On the other hand, the phase III CLOCC (Chemotherapy + Local Ablation Versus Chemotherapy) trial showed that the addition of local therapy of liver metastases by radiofrequency ablation with or without resection to systemic therapy significantly prolonged median overall survival from 40.5 to 45.6 months among 119 patients with colorectal cancer. Importantly, the allowed number of liver lesions was up to nine, and one-third of the study population had more than five metastases [8].

Although the situation in colorectal cancer is rather unique in that the liver is the first location of metastatic disease due to the predominant dissemination through the portal system, a tentative interpretation of these four trials could be that relying solely on the number of metastases is not sufficient to define a true oligometastatic disease. Even though some false oligometastatic cases harbouring unrecognised micrometastases derive a survival advantage from local ablation of all visible lesions, which in principle is a substitute for cytoreduction, it cannot be excluded that the crucial part of this benefit is conveyed by the systemic treatment which the patients receive in parallel or afterwards. Consequently, treating a false oligometastatic disease with local ablation may ultimately be harmful because of possible procedural complications, particularly in the case of invasive methods, leading to a prolonged interruption of systemic treatment or interfering with its initiation. The SABR-COMET trial noted a 20% increase in grade 2 or worse adverse events in the interventional arm. There were also three treatment-related deaths (4.5%) but none in the standard-of-care arm [6]. Hence, the need for correct identification of patients with a true oligometastatic disease seems warranted. At the same time, we acknowledge the existence of specific situations in oncology (e.g. oligoprogression and oligopersistence explained further in the text) where a strict distinction between a true and false oligometastatic disease may be marginal. These outliers should always be judged individually taking into account the patient’s preference, symptomatology, comorbidities and available treatment alternatives.

However, even if focusing on a true oligometastatic disease, we should keep in mind that the metastatic competence of malignant tumours is progressively increasing, influenced by many factors. The investigators of the prospective longitudinal cohort study TRACERx Renal (TRAcking renal cell Cancer Evolution through Therapy) analysed almost 1000 biopsies from 100 patients with metastatic clear-cell renal cell carcinoma and found that tumours initially presenting with an indolent disease course in the form of oligometastases gradually continued to progress towards a widespread phenotype [9]. Moreover, according to a retrospective study of different primary tumours, patients with a rate of new lung metastases below 0.6 per year live longer than those with a rate above 3.6 per year [10].

Taken together, it is the time factor that represents the key trait of a disease that can be cured by radical local therapy of all visible lesions. It is the time factor that defines the speed of tumour-cell shedding and proliferation. The slower cancer develops, the higher the chances of a local approach to succeed because of the widening therapeutic window-of-opportunity. A true oligometastatic disease therefore stands for slowly developing metastases, which we propose to call “argometastases” (from Greek “argos” meaning slow). But does terminology matter? A 2017 systematic review of seven studies covering several oncologic and non-oncologic conditions concluded that different terminology used for the same pathology impacts decision making [11]. Although the term “oligometastatic” was not explored in that study, we assume that the conclusions pertain to it as well. Moreover, oligometastatic presentation is rare, and it is well known that misdiagnosis and late diagnosis rank among the most important issues of rare diseases [12, 13]. In this respect, given the intrinsic feature of a number of lesions, some physicians facing patients with few metastases may be automatically tempted to propose local ablation if this is technically feasible. However, technical feasibility does not equal clinical relevance, the latter of which means to recognise a true oligometastatic disease. Its optimal definition will probably only be possible if biological characteristics, including genetic determinants, epigenetic modifiers and immune response markers, are integrated [14]. At present, despite continuous advances in this field, we are still far from their adoption in clinical practice.

Therefore, we would like to point out and summarise clinical findings which can be used to optimise the use of local ablation in patients presenting with newly diagnosed metastases. These recommendations do not cover situations where patients have disseminated cancer overall controlled by a systemic treatment except for several progressing lesions (oligoprogression) which can be easily treated for example by SABR. Neither will be discussed the consolidation of a few persisting metastases after otherwise successful systemic treatment (oligopersistence).

We have classified clinical findings associated with a true oligometastatic disease into three categories encompassing a low probability of occult metastases, low tumour growth rate and low tumour burden (Table 1). Disease-free interval is one of the major determinants of occult disease [15]. Several retrospective analyses demonstrated a positive predictive value of a longer disease-free interval after primary treatment for overall survival [16–18]. Due to the stochastic nature of this relationship, there is no cut-off to define the presence or absence of occult disease, and we expect the probability distribution to be continuous. Accordingly, a synchronous manifestation (de novo oligometastases) has a worse prognosis than a metachronous manifestation (oligorecurrence), which occurs after at least 3–6 months have elapsed since primary treatment [19]. The probability of occult dissemination also increases with the development of every new visible metastasis [15]. This corresponds to the observation that the lower the number of metastases, the better the prognosis with the best outcomes seen in patients with a single distant lesion [16, 17]. Analogously, a controlled primary tumour is a prerequisite for controlled cancer cell shedding, admitting that distant metastases can themselves be a source of further spread.

**Table 1:** Clinical characteristics of a true oligometastatic disease (“argometastases”).

**Low probability of occult metastases**
Metachronous presentation with a long disease-free interval^a^
Controlled primary tumour
No suspicious micronodules of unknown origin^b^
No regional lymph nodes involvement at initial diagnosis
Favourable distant organ site involvement^c^
Possibility to lower the detection threshold by auxiliary imaging and laboratory methods^d^
Susceptible tumour origin (histotype)
**Low tumour growth rate**
According to tumour growth kinetics as per a series of follow-up imaging (if available)
**Low tumour burden**
Limited size and number of lesions and limited number of organ sites allowing a safe and complete local ablation^e^

^a^Here, disease-free interval is defined as the time between oligometastatic presentation and completion of previous anticancer therapy. The probability of occult metastases progressively declines with increasing disease-free intervals.

^b^Usually between 2 and 8 mm (corresponding to the so-called grey zone) and found in different organs, typically in the lungs.

^c^Not only in terms of tumour location that should allow a safe and complete ablation but also in terms of tumour type associated with a survival benefit of local ablation (e.g. colorectal cancer oligometastases in the liver or head and neck cancer oligometastases in the lungs).

^d^Auxiliary imaging includes for example PET/CT, particularly with new tracers such as PSMA-targeted PET/CT and auxiliary laboratory methods comprise tumour marker tests and potentially also a liquid biopsy.

^e^Taking also into consideration the location of lesions within a given organ site.

The above-mentioned approach to disease-free interval should be distinguished from a situation when a disease-free interval (or a similar measure) is used to assess the efficacy of local ablation. While in the former case it evaluates the time period prior to local ablation in order to help identify true oligometastases and is usually recapitulated in clinical practice; in the latter case it not only looks at the time period after local ablation, being commonly employed in clinical trials, but can also be used in routine practice as feedback information because we expect a true oligometastatic disease not to recur after successful local ablation. The latter approach has one more implication in that it provides prevalence estimates. As an example, 5- and 10-year disease-free survival rates after liver metastasectomy in colorectal cancer patients are 25 and 20%, respectively. Keeping in mind that liver dissemination occurs in about half of these patients and only a minority of them undergo resection, these data confirm the rarity of a true oligometastatic phenotype [20].

Regional lymph node involvement at initial diagnosis is another negative predictive factor for subclinical hematogenous dissemination, particularly in the case of synchronous oligometastases but probably also in the metachronous setting [17, 19, 21]. The impact of primary tumour origin and histology is well known with some cancer types (e.g. colorectal cancer or clear-cell renal cell carcinoma) drawing more benefit from local treatment than the other [1, 22]. However, the phenotypic intertumoral heterogeneity is considerable and still not sufficiently understood as testified by the emerging concept of oligometastases in diseases traditionally considered typical examples of leukaemia-like dissemination like pancreatic cancer [23]. Moreover, although little is known about the role of organ tropism in the development of a true oligometastatic disease, the site of metastatic outgrowth seems to impact the success rates of local ablation as documented by different outcomes in patients with colorectal cancer and liver involvement (CLOCC trial) or lung involvement (PulMiCC trial) [7, 8]. Another example is head and neck cancer, where long-term survivorship after distant recurrence has been linked to human papillomavirus (HPV)-positive oropharyngeal carcinoma with lung oligometastases [24]. There are several factors that can explain these observations. Apart from a possible bias induced by a retrospective collection of data, rarity of some metastatic manifestations, cross-trial comparisons and differences in technical feasibility and preferred modalities according to anatomic locations, growing evidence suggests an implication of the microenvironment, particularly the immune system [25].

Perhaps the greatest potential for detecting micrometastases have imaging and laboratory methods. Currently, the detection threshold of the former modalities is about 2 mm, but such small lesions are non-specific. Usually, a size of about 8 mm triggers further investigations to conclude on their origin, either by means of imaging methods and/or bioptically (Fig. 1) [26]. The tissue sample is almost always mandatory to differentiate the original tumour from second primaries and non-malignant conditions. The presence of suspicious nodules in the grey zone between 2 and 8 mm poses a diagnostic challenge and prevents certainty in excluding occult metastases unless a biopsy is performed, which on the other hand may require more invasive interventions to obtain the tissue possibly accompanied by increased risk of complications (e.g. a pulmonary wedge resection).

**Fig. 1:**
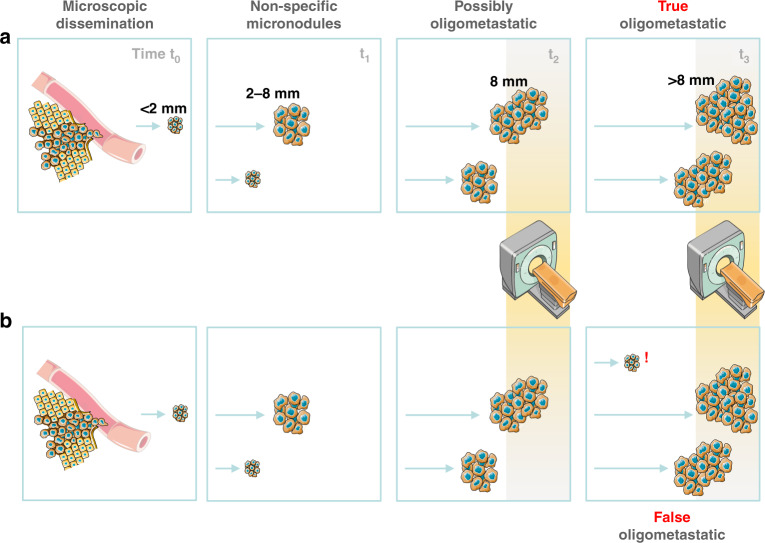
A simplified model of distant dissemination showing the difference between a true and false oligometastatic disease. In both scenarios (**a**, **b**), cancer cell shedding starts at time t_0_ leading to a detectable metastatic outgrowth at time t_1_. Lesions of at least 8 mm in diameter appearing at time t_2_ are amenable to a proper diagnostic workup including radiology, nuclear medicine and pathology. However, smaller leasions (2–8 mm) are often non-specific, requiring thus follow-up. Although at time t_3_, an oligometastatic state can be confirmed in both scenarious radiologically, only situation A corresponds to a true oligometastatic disease because there are no non-specific micronodules in the grey zone (2–8 mm) and, more imporantly, not any unrecognised subclinical dissemination. Figure includes modified templates from Servier Medical Art.

Imaging plays a decisive role in assessing growth kinetics, based on a chronological series of examinations, tumour burden, defined by size (volume) and number of lesions and number of organ sites, and location of lesions within a given organ site. All three parameters are inherently connected and determine the technical feasibility and safety of local treatment. Notably, tumour doubling time varies both on a case-by-case basis and in the same patient. According to volumetric analyses, it ranges from less than one week to more than 1 year, albeit usually being in the order of several months [27]. At an individual level, growth curves follow most accurately a Gompertzian model. Initial exponential size increments characterised by constant doubling times progressively slow down with the tumour becoming larger [28]. Taking this into account, a follow-up imaging to evaluate growth kinetics or assess the nature of suspicious lesions in the grey zone may be justified in selected patients but should always be carefully considered. A new promising method for improving the detection of metastatic lesions is prostate-specific membrane antigen (PSMA)-targeted PET/CT. The recent randomised phase II ORIOLE trial showed that if all PSMA-positive lesions are treated with SABR, the proportion of prostate cancer patients developing new metastases at 6 months is significantly lower than if some lesions are left untreated (16% versus 63%, *P* = 0.006) [29]. Hence, tailoring imaging modalities to tumour types is one of the promising avenues for future research.

Laboratory methods comprise both traditional tumour marker tests which have been validated in some malignancies, such as prostate-specific antigen (PSA) in prostate cancer, and presently still investigational liquid biopsies based on detection of different elements such as cell-free circulating tumour DNA, circulating tumour cells, microRNA or exosomes in body fluids, typically in the peripheral blood [30]. According to the PREDATOR study, postoperative analysis of molecular residual disease by circulating tumour DNA testing significantly correlates with disease-free survival in metastatic colorectal cancer patients undergoing metastasectomy with curative intent [31]. Data on pre-interventional liquid biopsy are still limited but could potentially contribute to quantification of disease burden and measurement of disease kinetics (e.g. circulating tumour DNA doubling time) [32, 33].

Our hypothetical model has several limitations some of which have already been addressed, especially a lack of prospective validation. Besides that, a restricted insight into tumour biology prevents us from integrating the multifaceted effects of heterogeneous behaviour of the primary tumour and its different metastases in terms of cancer cell shedding and proliferation and those of an outstanding phenomenon known as a dormant state allowing cancer cells to preserve their tumour-generating capacity and reawaken several years later [34]. Accumulating data confirm that an oligometastatic stage is a dynamic process, and evolutionary trajectories of malignant dissemination can even be bidirectional as shown in a preclinical study in which the investigators managed to reverse a polymetastatic to oligometastatic phenotype by epigenetic manipulations using microRNAs [35]. In this respect, patient outcomes differ according to exposition to different systemic drugs, including conventional chemotherapy, targeted agents and modern immunotherapy. Potentially impacting on characteristics and behaviour of oligometastases, these drugs can be given at various time points in the disease course, including but not limited to the above-mentioned scenarios of oligoprogression and oligopersistence. Finally, we acknowledge the fact that due to its multiparametric complexity, determining a true oligometastatic state with currently available diagnostic tools may be impossible in some cases. In such situations, the therapist’s expertise remains crucial, and decisions can be guided for example by local tumour growth imminently threatening to cause symptoms or lead to a missed opportunity for local ablation. In the same way, the risk of serious adverse events, either existing or impending, has a profound influence on the treatment choice. Nevertheless, with the advent of new technologies in clinical practices the gap of uncertainty will be undoubtedly getting narrower.

In conclusion, when employing local treatments in patients with few metastases, tumour dynamics seems to be the major denominator of therapeutic success. Cancers with slow tumour-cell shedding and slow proliferation leave a sufficiently broad window-of-opportunity to detect and treat accessible lesions. In case sporadic micronodules later develop in overt metastases, the indolent behaviour of such tardily appearing “argometastases” gives us another fair opportunity to eradicate them. A scientific terminology is a mighty tool that may eventually steer our decision making, not only in daily practice but also when dealing with a rare and sometimes over-diagnosed entity as a true oligometastatic disease probably is.

## Data Availability

The data supporting the findings of this paper are available from the corresponding author on request.
